# Limited Effect of Indolamine 2,3-Dioxygenase Expression and Enzymatic Activity on Lupus-Like Disease in B6.Nba2 Mice

**DOI:** 10.3389/fimmu.2019.02017

**Published:** 2019-08-27

**Authors:** Laura M. Davison, Jessica C. Liu, Lei Huang, Thomas M. Carroll, Andrew L. Mellor, Trine N. Jørgensen

**Affiliations:** ^1^Cleveland Clinic Foundation, Department of Immunology, Lerner Research Institute, Cleveland, OH, United States; ^2^Department of Molecular Medicine, Cleveland Clinic Lerner College of Medicine of Case Western Reserve University, Cleveland, OH, United States; ^3^Cancer Immunology, Inflammation and Tolerance Program, Georgia Cancer Center, Augusta University, Augusta, GA, United States

**Keywords:** antibodies, autoimmunity, dendritic cells, rodent, immunization, systemic lupus erythematosus, transgenic/knockout mice

## Abstract

B6.Nba2 mice spontaneously develop a lupus-like disease characterized by elevated levels of serum anti-nuclear autoantibody (ANA) immune complexes and constitutive type I interferon (IFNα) production. During disease progression, both plasmacytoid dendritic cells (pDCs) and antibody secreting plasma cells accumulate in spleens of B6.Nba2 mice. Indoleamine 2,3-dioxygenase (IDO) has been suggested to play a role in several autoimmune diseases including in the MRL/lpr model of mouse lupus-like disease; however, it remains unknown if IDO is involved in disease development and/or progression in other spontaneous models. We show here that IDO1 protein and total IDO enzymatic activity are significantly elevated in lupus-prone B6.Nba2 mice relative to B6 controls. IDO1 expression was restricted to PCs and SignR1^+^ macrophages in both strains, while significantly increased in B6.Nba2-derived SiglecH^+^ (SigH^+^) pDCs. Despite this unique expression pattern, neither pharmacologic inhibition of total IDO nor IDO1 gene ablation altered serum autoantibody levels, splenic immune cell activation pattern, or renal inflammation in B6.Nba2 mice. Interestingly, IDO pharmacologic inhibition, but not IDO1 deficiency, resulted in diminished complement factor C'3 fixation to kidney glomeruli, suggesting a possible therapeutic benefit of IDO inhibition in SLE patients with renal involvement.

## Introduction

Oxidative tryptophan (Trp) degradation is driven via the kynurenine pathway by one of three rate limiting enzymes: tryptophan 2,3-dioxygenase, indoleamine 2,3-dioxygenase (IDO)-1, or IDO2 (1). Induction of IDO activity depletes the local environment of Trp, triggering the general amino acid control non-depressible 2 (GCN2) stress pathway preventing T cell proliferation and inducing anergy (2–5). Concomitantly, downstream metabolites of IDO enzymatic activity such as 3-hydroxyanthranilic acid (3-HAA) and quinolinic acid (QA) have been shown to induce the generation, and support the maintenance, of regulatory T cells (Tregs) (6, 7). Thus, IDO (−1 and −2) have been suggested to mediate immune tolerance under various conditions such as chronic inflammation, cancer, and autoimmunity (8–11). IDO1 and IDO2 molecules have been suggested to be expressed by plasmacytoid dendritic cells (pDCs) (CD19^+^ and SigH+), macrophages (SignR1^+^), myeloid-derived suppressor cells (MDSCs), and lately also B cells and plasma cells (12–23). In addition to a role in T cell differentiation and activity, recent studies have suggested that IDO1 and/or IDO2 may also control humoral immunity via B-cell intrinsic mechanisms (17, 24).

Given this potent immunomodulatory function, IDO has been studied in patients with autoimmunity and in a variety of autoimmune mouse models. These studies found that total IDO is elevated in patients with rheumatoid arthritis (RA) (25) and systemic lupus erythematosus (SLE) (26–28). Similarly, IDO1 is overexpressed in spleens from MRL^lpr^ lupus-prone mice (18). Interestingly, inhibition of total IDO enzymatic activity via treatment with 1-d-MT, a tryptophan analog, accelerated the development of anti-nuclear autoantibodies (ANA) and glomerulonephritis (GN) in MRL^lpr^ lupus-prone mice (18). Likewise, in a model of experimental autoimmune encephalomyelitis (EAE), IDO1-deficiency exacerbated disease development by reducing numbers of Tregs, while treatment with the Treg-inducing metabolite 3-HAA ameliorated disease development (6). Similarly, collagen-induced arthritis and TNBS-induced colitis models also showed exacerbated disease development after 1-MT treatment (29, 30) and it has been suggested that IDO1-deficient mice responded stronger to T-independent antigen immunization than wild type non-autoimmune mice (17). Oppositely however, IDO1-deficiency had no effect on the spontaneous development of experimental RA in transgenic KRN mice, although treatment with 1-d/l-MT or IDO2-deficiency resulted in less severe disease (31, 32). Thus, IDO1 and IDO2 may play different roles in different disease models.

B6.Nba2 mice develop a lupus-like disease evident by the presence of hypergammaglobulinemia, elevated serum ANA, increased type I interferon (IFNα) levels, splenomegaly, GN, and IgG-immune complex (IgG-IC) deposition in the kidneys (33–37). Already at 2 months of age, and increasing thereafter, B6.Nba2 mice present with hyperactivated B cells and elevated levels of plasma cells and pDCs as compared with age- and sex-matched B6 mice (36, 37). Given the potential role of IDO in autoimmunity and the accumulation of these potential IDO-producing cell subsets, we investigated the role of IDO in lupus-like disease development of progression in B6.Nba2 mice. We show here that the levels of IDO1 protein and IDO enzymatic activity were in fact significantly elevated in spleens of B6.Nba2 mice. In agreement with previous literature, IDO1 protein was measurable in SigH^+^ pDCs, SignR1^+^ macrophages as well as plasma cells, but not in T cells or B cells. Surprisingly, however, neither IDO1-deficiency nor pharmacological inhibition of IDO enzymatic activity had any effect on disease parameters including serum ANA, splenomegaly, hyperactive lymphocytes, GN, and IgG-IC deposition. On the other hand, pharmacological inhibition alone resulted in diminished complement factor C'3 fixation to kidney glomeruli. Dual manipulation (1-d-MT treatment of IDO1^−/−^ B6.Nba2 mice) had no effect on spontaneous lupus-like disease development, despite elevating the humoral immune response to T-dependent antigen. Thus, we conclude that IDO suppresses humoral immunity to exogenous antigen in lupus-prone B6.Nba2 mice but fails to affect lupus-like disease development. Yet further studies will be needed to determine whether IDO inhibition could be beneficial for SLE patients with renal involvement.

## Materials and Methods

### Mice

C57BL/6J (B6) mice were purchased from The Jackson Laboratory (Bar Harbor, ME). B6.Nba2 mice were transferred from the University of Colorado at Denver Health Sciences Center and are currently bred in our facility. B6.Nba2 IDO1-deficient mice (B6.Nba2.IDO1^−/−^) were generated in house by backcrossing B6.IDO1^−/−^ (generously provided by Dr. Ram Nagaraj of Case Western Reserve University) onto the B6.Nba2 background. Marker-assisted polymerase chain reaction technique was employed and genotypes were ascertained to ensure that the complete *Nba2* region was maintained, as previously described (36, 37). Genotyping for *Ido1* was performed according to the protocol by Jackson Laboratories. All mice were maintained in the Biological Research Unit at the Lerner Research Institute, in accordance with Cleveland Clinic Foundation Animal Research Committee guidelines. Animal studies were approved by the Institutional Animal Care and Use Committee of the Lerner Research Institute of the Cleveland Clinic Foundation and conducted in compliance with guidelines issued by the National Institutes of Health.

### Systemic IDO Inhibition With 1-d-MT

Systemic inhibition of total IDO was achieved using 1-d-MT, as described (38). Briefly, mice were treated with 2 mg/mL 1-d-MT (in 8 mM NaOH, pH 7.0 ± 0.2), in the drinking water for 4–20 consecutive weeks, beginning at 8–12 weeks of age. Control mice were given sham water (8 mM NaOH, pH 7.0 ± 0.2). Water bottles were kept in the dark and new water was supplied at least weekly.

### Flow Cytometry

Splenic single cell suspensions were prepared by gently separating single cells between the frosted areas of two microscope slides. Red blood cells were lysed using 1x ACK buffer (0.15 M NH_4_Cl, 0.01 M KHCO_3_, 0.1 mM EDTA). Cells were stained for detection of surface and intracellular antigens using antibodies specific to B220, CD3, CD4, CD5, CD8, CD11b, CD11c, CD19, CD21/35, CD22, CD23, CD25, CD38, CD40, CD44, CD62L, CD69, CD86, CD93 (AA4.1 clone), CD115, CD138, F4/80, Foxp3, GL-7, Gr1, ICOS, IgD, IgM, MHCII, PD-1, PDCA-1, Siglec-H, (eBiosciences Inc, CA), MOMA-1 (Abcam, Cambridge, MA), CXCR5, Ly6-G (BD Biosciences, CA), SignR-1 (AbD Serotec, NC). All samples were treated with unlabeled anti-CD16/32 antibodies to reduce Fc-receptor dependent non-specific antibody binding. Analyses of Foxp3 expressing cells were performed by preparing the samples for intracellular staining using the manufacturer's protocol (Foxp3/Transcription Factor Staining Buffer Set, eBiosciences). Non-specific, fluorescently-conjugated rat IgG2a antibody (eBiosciences) was used as a control. Samples were run on a FACS Calibur (BD Biosciences, San Jose, CA) and data analysis was performed using FlowJo^TM^ (Tree Star Inc., OR) version 9.8.2. Live cells were determined based on forward and side scatter properties; individual cell subsets were determined by marker positivity, as indicated under each population name in Table 1.

**Table 1:** Splenic cellular composition in 12 week old B6, B6.Nba2, and IDO-manipulated B6.Nba2 mice.

	**B6^a^**	**B6.Nba2^b^**	**B6.Nba2.IDO1^–/–c^**	**B6.Nba2+ 1-D-MT^d^**	**B6.Nba2.IDO1^**–/–**^**+** 1-D-MT^e^**
Total splenocytes in millions	75 ± 8.6^f^	130 ± 15.6^*^	113 ± 11.8^*^	100 ± 8.16^(*)^	117 ± 23.1
**DENDRITIC CELLS (%)**^g^					
cDCs	0.8% ± 0.02	1.1% ± 0.05^**^	1.1% ± 0.17	0.9% ± 0.09	0.9% ± 0.08
pDCs (all)	0.8% ± 0.01	1.4% ± 0.17^**^	1.3% ± 0.14^*^	1.1% ± 0.23	1.1% ± 0.23
CD19^+^ pDCs	0.4% ± 0.05	0.9% ± 0.19^*^	1.1% ± 0.13^*^	0.7% ± 0.19	0.9% ± 0.23^(*)^
SiglecH^+^ pDCs	0.3% ± 0.04	0.2% ± 0.02	0.2% ± 0.02	0.2% ± 0.01	0.2% ± 0.02
**T Cells (%)**					
CD8^+^(all)	14.1% ± 0.49	11.8% ± 0.62^*^	10.5% ± 1.40^(*)^	13.0% ± 0.41	11.3% ± 1.08^(*)^
CD4^+^(all)	22.4% ± 1.83	22.2% ± 1.92	18.7% ± 1.99	22.7% ± 1.68	18.7% ± 1.99
Foxp3^+^CD4^+^Treg	1.1% ± 0.21	1.5% ± 0.13	1.3% ± 0.10	1.4% ± 0.14	1.3% ± 0.05
Naïve CD4^+^CD62L^hi^CD44^low^	15.9% ± 1.2	10.7% ± 1.44^*^	8.8% ± 2.05^*^	12.8% ± 1.49	8.6% ± 1.62^*^
Eff-mem CD4^+^CD62L^low^CD44^hi^	2.8% ± 0.17	6.1% ± 0.63^**^	6.0% ± 0.40^**^	4.7% ± 0.40^**^	5.8% ± 0.59^*^
CD69^+^CD4^+^	1.6% ± 0.16	2.9% ± 0.38^*^	2.8% ± 0.33^*^	2.2% ± 0.21^(*)^	2.6% ± 0.21^*^
**MYELOID CELLS (%)**					
Gr1^+^CD11b^+^	2.9% ± 0.41	2.6% ± 0.26	5.3% ± 1.83	2.6% ± 0.33	2.7% ± 0.59
F4/80^+^CD11b^+^	1.2% ± 0.10	0.6% ± 0.07^**^	0.6% ± 0.25	0.5% ± 0.05^**^	0.5% ± 0.06^**^
SignR1^+^MΦ	0.5% ± 0.01	0.3% ± 0.03^***^	0.3% ± 0.06^*^	0.3% ± 0.04^*^	0.2% ± 0.01^***^ ^(#)^
**B Cells (%)**					
B cells (all)	48.1% ± 3.58	49.4% ± 2.64	49.5% ± 2.86	49.9% ± 2.53	53.1% ± 2.58
CD69^+^B220^+^	0.1% ± 0.02	0.2% ± 0.03^*^	0.2% ± 0.01^*^	0.2% ± 0.01	0.2% ± 0.03
GC B cells	0.5% ± 0.08	1.9% ± 0.29^**^	1.5% ± 0.10^**^	1.5% ± 0.30^*^	1.4% ± 0.19^*^
CD138^+^B220^low^ plasma cells	0.1% ± 0.002	0.6% ± 0.13^**^	1.0% ± 0.16^*^	0.5% ± 0.12^**^	0.5% ± 0.11^*^

a n = 3;

b n = 8;

c n = 4;

d n = 7;

e n = 4;

f AVG ± SEM, numbers expressed in millions.

g *AVG ± SEM, numbers represent % of total splenocytes*.

(^*^) *p = 0.05-0.09*.

* p < 0.05;

** p < 0.01;

*** *p < 0.001, vs. B6*.

(#) *p = 0.05–0.09 vs. B6.Nba2*.

### Western Blotting

Western blot was performed using total splenocyte lysates. A single cell suspension was prepared from total spleen samples, red blood cells were lysed, and remaining cells were resuspended in Cell Lysis Buffer (Cell Signaling) containing 10% PhosSTOP (Roche Applied Science). Lysates were denatured by boiling for 5 min in Laemli sample buffer (60 mM Tris-HCl, pH 6.8, 2% SDS, 10% glycerol, 0.01% bromophenol blue, β-mercaptoethanol) and 15 μg of protein was loaded onto a 1.0 mm-thick 12% SDS-PAGE gel. Gel proteins were transblotted onto nitrocellulose membranes (0.45 μm; Amersham Biosciences, Germany). The membranes were blocked with phosphate buffered saline (PBS: 137 mM NaCl, 2.7 mM KCl, 10 mM Na_2_HPO_4_, 1.8 mM KH_2_PO_4_; pH 7.4) containing 0.05% Tween-20 (PBST) and 2% non-fat dry milk for 1 h at room temperature. Membranes were incubated overnight at 4°C in PBST containing 2% non-fat dry milk with rabbit polyclonal IDO-specific antibody (1:1,000, US Biological, MA). Membranes were washed in PBST, incubated with horseradish peroxidase-conjugated anti-rabbit IgG (1:4,000, Santa Cruz Biotechnology, TX) for 1 h at room temperature, and washed again, before being developed using Amersham ECL Western Blotting Detection Reagents (GE Healthcare, Buckinghamshire, UK). Bands were detected after exposure to Amersham Hyperfilm ECL high performance chemiluminescence film (GE Healthcare) and quantified by densitometry using ImageJ software (version 1.42q, NIH). After quantification of IDO bands, gels were stripped in stripping buffer (2.5 mM Tris-HCl, pH 6.7, 100 mM β-mercaptoethanol, 2% SDS) and levels of actin were determined as above using goat polyclonal anti-Actin(C-11): sc-1615 (at 0.2 ug/mL; Santa Cruz Biotechnology, TX). Results are expressed as ratios of intensity of IDO1 to β-Actin internal standard.

### IDO Enzymatic Activity Assay

Detection of total IDO enzymatic activity was performed as previously described (39, 40). Briefly, spleen tissue was flash frozen on dry ice and kept at −80°C until further processing. Tissues were then homogenized with a Polytron homogenizer (Kinematica, Lucerne, Switzerland) in 1.5x volume of ice-cold buffer (0.14 M KCl, 20 mM potassium phosphate buffer, pH 7.0). Homogenized samples were centrifuged for 10 min at 7,000 × g and 4°C. Aliquots of supernatant were taken for IDO enzymatic activity measurement and mixed 1:1 in substrate solution (100 mM potassium phosphate buffer, pH 6.5; 50 μM methylene blue; 20 μg catalase; 50 mM ascorbate; 0.4 mM l-Trp) in a total volume 100 μL. Reaction mixture was incubated at 37°C, then acidified with 3% perchloric acid and centrifuged as before. The concentrations of enzymatic products were measured by HPLC. Enzyme activity is expressed as product content per hour per gram of tissue protein.

### Immunization

On day 0, mice were immunized intraperitoneally with 20 μg (4-hydroxy-3-nitrophenylacetyl)27 conjugated chicken γ-globulin (NP_27_-CGG) in CFA (Sigma-Aldrich, St. Louis, MO) in a total volume of 200 μl per mouse. Serum samples were collected on days -1, 7, 14, 21, and 28 by tail-vein bleeding. At day 28, all mice were sacrificed and splenic cell populations were determined by flow cytometry (see above). Treatment with 1-d-MT was initiated at the time of immunization and maintained throughout the study.

### Measurement of Serum Antibodies by ELISA

Murine anti-chromatin-IgG, anti-histone-IgG, total IgG, and total IgM antibodies were measured as previously described (33). Sera were diluted in serum diluent (5 mg/ml bovine γ-globulin, 5% gelatin, 0.05% Tween in PBS) at 1:100,000 for determination of total IgG and IgM levels, and at 1:300 for antigen-specific antinuclear antibodies. Anti-NP specific antibodies were determined by ELISA as previously described (41), at a dilution of 1:10,000. Horse-radish peroxidase conjugated secondary antibodies recognizing mouse IgG, IgG_1_, or IgG_2a_ heavy chains (Southern Biotech, AL) were used at 1:2,000, 1:2,000, and 1:1,000 dilutions, respectively. Colorimetric readings were obtained using a Victor 3 plate reader (Perkin Elmer, Waltham, MA).

### Immunofluorescence Staining

Kidney samples were embedded in Tissue-Tek optimal cutting temperature compound (Sakura) and snap-frozen. Sections (5 μm) were air-dried, fixed with cold acetone, and washed in PBS buffer. To prevent non-specific binding, sections were blocked with anti-mouse CD16/CD32 (1:200, eBioscience) in 10% non-immune goat serum (Invitrogen). Sections were stained with Tx Red-conjugated anti-mouse IgG2b (1:500, Southern Biotech) and FITC-conjugated goat anti-mouse C3 (1:500, ICL). Sections were then washed and mounted with 70% glycerol. Image processing and analysis was performed on the BZ-X700 all-in-one fluorescence microscope (Keyence, Osaka, Japan).

### Statistical Analyses

Statistical differences between cell populations and protein levels of IDO1 were calculated using the Student's unpaired *t*-test (with Welch's correction for cell populations). Differences in longitudinal serum antibody data were determined using two-way ANOVA test. For single time point analysis of serum antibody levels, differences were calculated using the Student's unpaired *t*-test with Welch's correction. For all analyses, *p* < 0.05 was considered statistically significant.

## Results

### IDO1 Is Elevated in B6.Nba2 Lupus-Prone Mice

**Figure 1:**
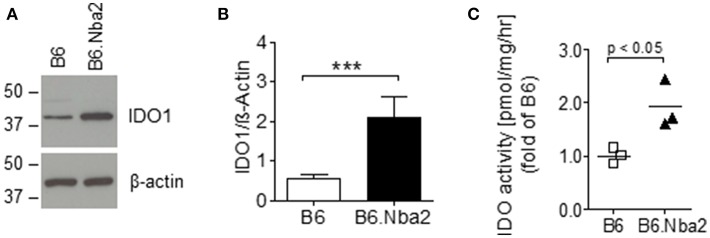
IDO1 is elevated in disease-prone B6.Nba2 mice. Western blot analysis of splenic IDO1 levels in unmanipulated, 16 week old female B6 and B6.Nba2 mice. **(A)** One sample representative is shown for B6 and B6.Nba2. **(B)** Quantification of IDO1 western blot analyses [B6: *n* = 6; B6.Nba2: *n* = 3]. Each bar shows Mean ± SEM. Student's unpaired *t*-test: ***p < 0.001. **(C)** Analysis of IDO enzymatic activity in spleens of B6 and B6.Nba2. Each symbol represents data from an individual mouse. Shown is Mean ± SEM. Student's unpaired *t-*test: *p* < 0.05.

Abnormal levels of IDO have been reported in both SLE patients and mouse models of lupus (18, 28). To establish if IDO was dysregulated in lupus-prone B6.Nba2 mice, we tested basal levels of IDO1 and IDO2 in spleens from unmanipulated female B6.Nba2 strains and control age- and sex-matched B6 mice. IDO1 levels were significantly elevated in spleens of female B6.Nba2 mice (Figures 1A,B). We were unable to detect IDO2 protein in total spleen samples from these mice as previously described (42) (data not shown). Since levels of IDO1 protein are not necessarily a direct indication of total IDO enzymatic activity, quick-frozen spleen samples were subsequently tested for total IDO enzymatic activity in an *in vitro* tryptophan (Trp) catabolism assay (40). Enzymatic Trp degrading activity was significantly elevated in female B6.Nba2 mice as compared with age- and sex-matched B6 control mice (Figure 1C). In conclusion, both levels of IDO1 and total IDO enzymatic activity were found to be elevated in spleens from lupus-prone B6.Nba2 mice.

### IDO1 Deficiency and Total IDO Enzymatic Inhibition Fail to Affect ANA Levels in B6.Nba2 Mice

**Figure 2:**
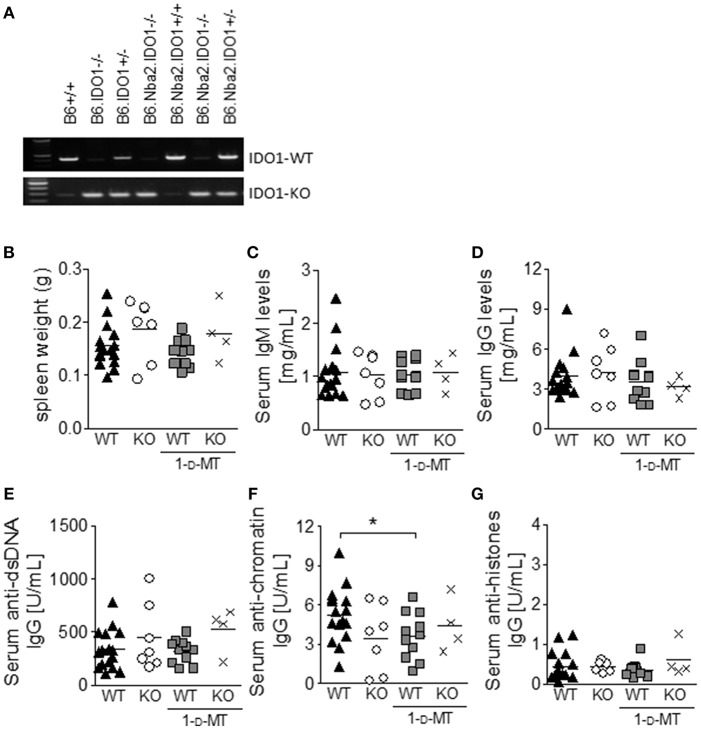
IDO1-deficiency and/or 1-d-MT treatment do not affect the development of splenomegaly and autoantibody production in B6.Nba2 mice. IDO1-deficient and –sufficient B6.Nba2 mice were bred **(A)** and treated with PBS or 1-d-MT in the drinking water from 8 to 24 weeks of age. At 24 weeks of age, mice were harvested and spleen weights were recorded **(B)**. Serum was obtained monthly and evaluated for total IgM **(C)**, total IgG **(D)**, anti-dsDNA IgG **(E)**, anti-chromatin IgG **(F)**, and anti-histone IgG **(G)**. Shown are data from 24 week old mice. Each symbol represents one mouse. *p < 0.05, Student's unpaired *t*-test with Welch's correction.

Previously, IDO-dependent Trp metabolism has been shown to play a role in autoimmune disease etiology (24, 26, 28). Given that IDO1 is both elevated and active in B6.Nba2 mice, we investigated if expression of IDO1 and/or total IDO enzymatic activity affected the production of autoantibodies in these mice. Cohorts of B6.Nba2.IDO1^−/−^ and B6.Nba2.IDO1^+/+^ littermates (Figure 2A) were treated with 1-d-MT or sham-treated and followed longitudinally until 24 weeks of age for the development of hyper IgM syndrome, hypergammaglobulinemia, and elevated serum ANA levels as previously described (34, 36). At the time of harvest, spleens were isolated and weighed for detection of splenomegaly. There was no effect of IDO1-deficiency or total IDO enzymatic inhibition on spleen size (Figure 2B). Serum total IgM or IgG antibody levels were neither significantly nor consistently affected by IDO1 deficiency and/or 1-d-MT treatment at any time point (shown are data from 24 week old mice; Figures 2C–G). Although B6.Nba2 mice treated with 1-d-MT showed statistically significantly lower levels of anti-chromatin IgG than untreated, IDO1-sufficient B6.Nba2 mice (Figure 2F), this observation was not consistent across other autoantibody specificities (Figures 2E,G).

Disease progression in B6.Nba2 mice is prominent from 8 to 16 weeks of age where splenic cellular differences first become measurable (35–37). In order to adequately determine an effect of IDO manipulation on early differences in autoantibody levels, we repeated the above experiment following mice from 8 to 16 weeks of age. Consistent with data from 24 week old mice, there were no changes in serum ANA levels in 12 week old mice after 4 weeks of IDO manipulation (Supplemental Figure 1).

### Manipulation of IDO Does Not Affect Immune Cell Abnormalities in B6.Nba2 Mice

Young B6.Nba2 mice display a number of characteristic immune cell abnormalities (35–37). We analyzed spleens from 12 week old B6.Nba2, IDO-manipulated B6.Nba2, and control non-autoimmune B6 mice for the presence and activation status of a number of immune cells involved in lupus pathogenesis. In correlation with previous data, unmanipulated B6.Nba2 mice had significantly elevated numbers of total splenocytes as compared with the B6 control strain (Table 1, top row; *p* < 0.05). The percentage of spleen cell subsets and the total number of cells within each subset present in spleens are presented in Table 1 and Supplemental Table 1, respectively. Overall, the percentages of total T cells and total B cells were not significantly different between B6 and B6.Nba2 mice at this age, except for a small reduction in the percentage of total CD8^+^ T cells in B6.Nba2 mice (*p* < 0.05; Table 1). In contrast, the percentages and numbers of conventional dendritic cells (cDCs) and plasmacytoid DCs (pDCs) were elevated in unmanipulated B6.Nba2 and IDO1^−/−^ B6.Nba2 mice (Table 1, *p* < 0.05–0.01). The increase in total pDCs in both strains was mainly due to an increase in the CD19^+^ pDC subpopulation (*p* < 0.05 compared with B6), while there were no significant differences in the percentages of SigH^+^ pDCs between any of the strains. Consistent with a disease-prone phenotype and previous findings (35, 37), we also identified an increased presence of activated lymphocytes (CD69^+^ T and B cells), a significant shift in the ratio between naïve and effector-memory CD4^+^ T cells in B6 vs. B6.Nba2, and an increase in the percentage of germinal center (GC) B cells and plasma cells, (*p* < 0.001, Table 1 and data not shown). Similarly, the percentages and numbers of CD69^+^ T and B cells were significantly increased in IDO^−/−^ B6.Nba2 mice, while only the percentage of CD69^+^ T cells was significantly elevated in 1-d-MT treated mice. The percentages of germinal center (GC) B cells and plasma cells were elevated in all B6.Nba2 mice regardless of the IDO status (*p* < 0.05–0.01; Table 1 and Supplemental Table 1). This pattern was further supported by the lack of statistically significant differences between unmanipulated B6.Nba2 and any of the IDO-manipulated groups. The observation was not an effect of the young age of these mice, as similar data were obtained from 24 to 28 week old mice (data not shown). Finally, the percentages, but not numbers, of F4/80^+^ macrophages and SignR1^+^ MZ macrophages were reduced in all B6.Nba2 mice (*p* < 0.05–0.001), while there were no differences in the percentages and numbers of Gr1^+^CD11b^+^ myeloid cells.

### Manipulation of IDO Enzymatic Activity in B6.Nba2 Mice Does Not Affect Renal Inflammation but Diminishes Complement Fixation in Kidney Glomeruli

**Figure 3:**
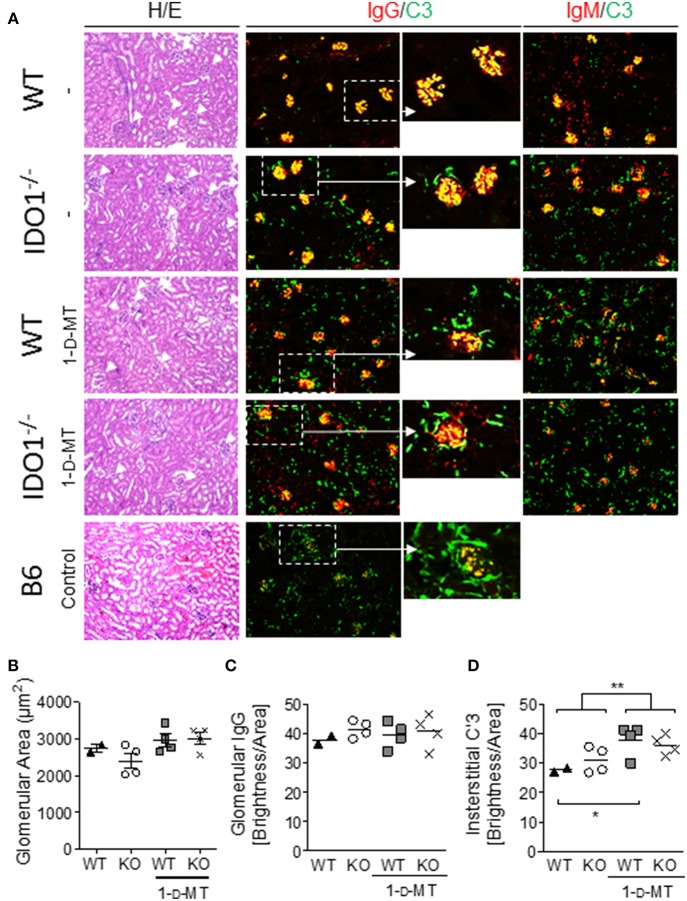
1-d-MT treatment does not affect renal inflammation but reduces complement factor C'3 fixation in kidney glomeruli. Kidneys were harvested from 16 week old IDO1-deficient and -sufficient B6.Nba2 mice treated with PBS or 1-d-MT and from female age-matched B6 control mice. Hematoxylin/eosin staining showed no significant differences in glomerulonephritis and mesangial proliferation **(A,B)**. Staining for IgG, and IgM showed no measurable differences in glomerular IgG and IgM brightness levels between B6.Nba2 mice (**C** and not shown) but a significant increase in interstitial C'3 brightness levels in 1-d-MT treated B6.Nba2 mice (**) **(A,D)**. Each symbol represents one mouse. *p < 0.05; **p < 0.01; Student's unpaired *t*-test with Welch's correction.

Evidence from us and others suggests that autoantibody levels and renal disease may not always correspond in animal models of lupus (33, 43, 44). We therefore determined the presence of GN, mesangial proliferation, IgG-, and IgM-IC deposition and subsequent complement fixation to kidney glomeruli in B6.Nba2, B6.Nba2 treated with 1-d-MT, B6.Nba2.IDO1^−/−^, and B6.Nba2.IDO1^−/−^ treated with 1-d-MT. Inflammation of kidneys from 12 to 24 week old mice was determined based on hematoxylin/eosin stainings. Low grade GN was observed in all groups of B6.Nba2 mice and neither IDO1 deficiency nor IDO1 enzymatic inhibition significantly affected renal inflammation (Figure 3A). Similarly, mesangial proliferation as measured by glomerular size, was unchanged between the groups of mice (Figure 3B). Secondly, we determined the presence of IgG, IgM, and complement factor C'3 in the kidneys by immunofluorescence staining. While there was no difference in the levels of IgG and IgM deposition among the groups of B6.Nba2 mice (Figure 3A, red stain; Figure 3C), we observed significantly increased interstitial-specific (and reduced glomerular-specific) complement factor C'3 staining in mice treated with 1-d-MT, regardless of the presence of IDO1 (*p* < 0.01; Figure 3A, green stain; Figure 3D). This pattern was similar to staining results obtained from age- and sex-matched B6 mice (Figure 3A, bottom row), suggesting that 1-d-MT treatment dampened renal complement factor C'3 fixation to glomeruli in an IDO1-independent manner.

### Inhibition of IDO Increases the T-Dependent Antibody Response to Exogenous Antigen

**Figure 4:**
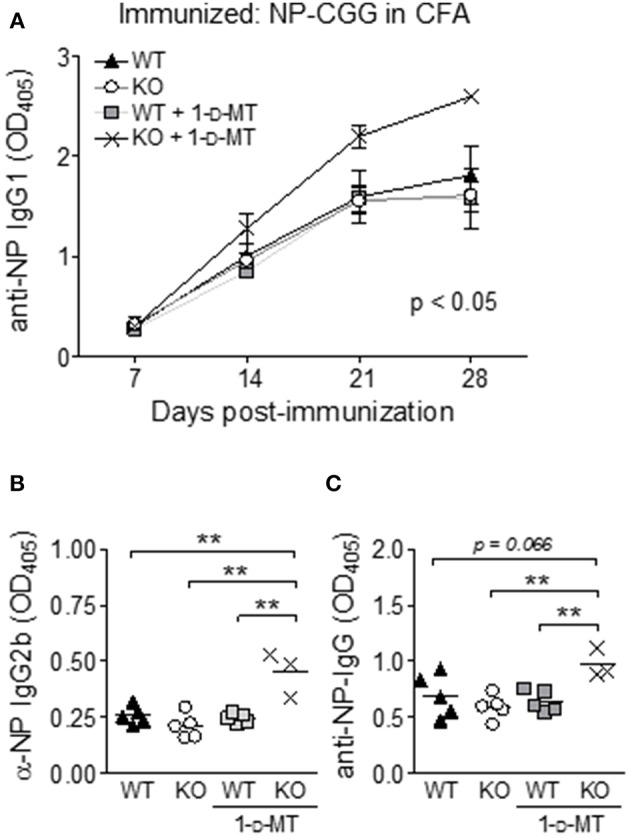
IDO suppresses antibody responses to an exogenous T-dependent antigen in B6.Nba2 mice. B6.Nba2 and B6.Nba2.IDO^−/−^ were left treated or not with 1-d-MT after immunization with 20 μg NP-CGG conjugate in Complete Freund's Adjuvant (CFA). **(A)** The data display the mean ± SEM of anti-NP IgG1 levels in serum obtained from three separate rounds of immunization; B6.Nba2 (*n* = 5), B6.Nba2 + 1-d-MT (*n* = 5), B6.Nba2.IDO1^−/−^ (*n* = 5), B6.Nba2.IDO1^−/−^ + 1-d-MT (*n* = 3). **(B,C)** Serum IgG2b and total IgG levels 28 days post immunization. Each symbol represents one mouse. **p < 0.01. Two-way ANOVA **(A)** or Student's unpaired *t*-test with Welch's correction **(B,C)**.

Previously IDO has been found to regulate the crosstalk between T and B cells in the K/BxN mouse model of rheumatoid arthritis (24). Thus, it was surprising that manipulation of IDO did not affect ANA levels in B6.Nba2 mice known to depend on both T and B cells (35, 45). To identify if IDO is involved in antibody-driven immunity to exogenous antigen in this model at all, B6.Nba2, B6.Nba2.IDO1^−/−^, B6.Nba2 mice treated with 1-d-MT, and B6.Nba2.IDO1^−/−^ mice treated with 1-d-MT were immunized with the T-dependent antigen NP-Chicken γ-Globulin (GCC) in Complete Freund's Adjuvant (CFA). Mice were immunized on day 0 and bled every 7 days thereafter to establish if IDO1-deficiency and/or inhibition of IDO affected the generation of NP-specific antibodies. As the anti-NP antibody response developed, 1-d-MT-treated B6.Nba2.IDO1^−/−^ mice displayed significantly elevated anti-NP specific IgG_1_ antibodies as compared with all other groups of mice (*p* < 0.05, Figure 4A). Neither IDO1-deficiency nor 1-d-MT treatment alone, however, had any effect on the antibody response. To ensure that this increase was not simply a result of a change in the cytokine balance and hence class switching properties, we also tested levels of anti-NP specific total IgG and IgG_2b_ antibodies and found that all isotypes were similarly elevated in 1-d-MT treated B6.Nba2.IDO1^−/−^ mice (Figures 4B,C). Thus, IDO1 deficiency coupled with pharmacologic inhibition of IDO enhanced the antibody response to exogenous T-dependent antigen in B6.Nba2 mice.

## Discussion

It has been speculated that IDO plays a regulatory role in SLE due to the identification of elevated IDO activity in SLE patients with an active type I interferon signature (28). We have previously reported that type I interferon-induced signaling and type I interferon-producing pDCs are required for disease development in B6.Nba2 mice (33, 37), prompting us to investigate the role of IDO in this model. Corresponding with a previous report in the MRL^lpr/lpr^ mouse model of lupus (18), we found elevated levels of IDO1 and increased total IDO enzymatic activity in spleen tissue from lupus-prone female B6.Nba2 mice as compared with non-autoimmune prone female B6 mice. We were unable to detect IDO2 protein in total spleen samples from either strain.

Expression of IDO1 by spleen cell subsets have previously been shown in SignR1^+^ MZM (18, 46) and SigH^+^ pDCs (22, 23). Unpublished data from our lab suggest a similar distribution pattern, although further studies are needed to verify these due to recently identified non-specific binding patterns by a subset of IDO1-specific antibodies (47). A functional role for IDO1 expression in these cell subsets, however, has been described. First, SignR1^+^ MZM are involved in the control of immune activation in response to the uptake and clearance of blood borne antigens via scavenger receptors MARCO, co-expressed by many but not all SignR1^+^ MZM, and SignR1 itself (48–52). In addition, MZM have been suggested to be involved in the clearance of apoptotic cells and the induction of self-tolerance (46). Thus, IDO1 expression by this highly specialized cell subset likely serves to dampen the activation of adaptive immunity under non-infectious conditions. Interestingly, we found reduced percentages of SignR1^+^ MZM in all B6.Nba2 mice as compared with B6 control mice (see Table 1), suggesting that SignR1^+^ MZM could play a role in disease pathogenesis in the B6.Nba2 model. Secondly, pDCs accumulate in B6.Nba2 mice (36) and disease progression can be halted by ablation of SigH^+^ pDCs, the latter potentially due to the ensuing reduction in type I interferon production (37). While expression of IDO1 by pDCs seems counterintuitive, IDO1-expressing SigH^+^ pDCs have been shown to play an immunoprotective role in animal models of multiple sclerosis and atherosclerosis (22, 23). Our preliminary data similarly suggest elevated IDO1 expression in SigH^+^ pDCs in both B6.Nba2 and lupus-prone (NZB x NZW)F1 mice. Whether IDO1 expression by pDCs is induced by IFNγ, type I interferon or both as previously described (12, 53), and therefore a secondary readout of heightened disease activity, remains under investigation.

Complement activation is a well-known indication of lupus-like renal disease in mouse models of SLE, although the precise mechanism by which complement is involved remains unclear. For example, genetic deficiency of DAF, a natural C′3 inhibitor, in lupus-prone MRL^lpr/lpr^ mice did not affect the severity of lupus nephritis (54), while overexpression of Crry, another C′3 inhibitor, protected against lupus nephritis in the same model (55). It is interesting to note, that despite IDO1/total IDO manipulation having no effect on autoantibody production, IgG-IC depositions within the kidney glomeruli, and spleen cell subset activation and accumulation patterns, we still observed that interstitial complement factor C'3 staining remained high upon treatment with 1-d-MT, similarly to the pattern observed in non-autoimmune B6 mice. Correspondingly, C'3 staining decreased within the glomeruli of 1-d-MT-treated B6.Nba2 mice. There is some evidence that IDO1 and/or IDO2 activity can affect complement activation in different diseases (56, 57), however the exact mechanism remains unknown and no study has specifically investigated the role of IDO on complement in renal disease. In fact, IDO1 has been found to play different roles in different renal diseases. In a nephrotoxic serum nephritis mouse model, IDO enzymatic inhibition using 1-MT (-d or -l isomer) exacerbated proteinuria and end-stage renal disease signifying a protective role for IDO (58), although whether this can be accredited to IDO1 or IDO2 was not determined. In a separate study, IDO1 expression and function was associated with inhibition of the GCN2 stress pathway leading to reduced macrophage inflammation and lower inflammatory cytokine levels (59). Oppositely, IDO1 was found to play a pathogenic role following renal ischemia-reperfusion injury, as IDO1 deficiency or total IDO enzymatic inhibition prevented kidney injury as determined by serum creatinine levels (60). In this study, the use of 1-d-MT vs. 1-d/l-MT or 1-l-MT was also not described, however the similarity of the results obtained from IDO1-KO mice and 1-MT treated mice, suggest that the effect was mediated by IDO. Finally, in chronic kidney disease (CKD) patients, total IDO enzymatic activity correlated with the severity of the disease; although it was speculated that this association was as a result of the ongoing inflammation, and thus not a primary, driving factor of CKD (61). Taken together, it is possible that inhibition of IDO (−1 or−2) via 1-d-MT treatment affects T cells, properdin levels and/or results in reduced levels of inflammatory cytokines by yet other mechanisms, ultimately leading to diminished macrophage infiltration and less complement activation.

In summary, we have here shown that B6.Nba2 lupus-prone mice express elevated levels of IDO1 and increased total IDO enzymatic activity. Given the elevated levels of IFNα reported in both B6.Nba2 and (NZB x NZW)F1 mice, it is possible that the elevated levels of IDO1 protein is due to a direct transcriptional effect, although future studies manipulating IFNα levels in B6.Nba2 mice are needed to firmly establish this association. Surprisingly, IDO1 expression and total IDO enzymatic activity were largely dispensable for lupus-like disease development in B6.Nba2 mice, as neither genetic ablation of IDO1 nor chemical inhibition of total IDO resulted in any consistent statistically significant changes in serum ANA levels or *Nba2*-driven splenic cell subset abnormalities, although significantly less C'3 was co-localized with IgG-IC within kidney glomeruli of mice treated with 1-d-MT. Future studies evaluating the effects of more specific IDO inhibitors on renal tissue are needed to establish if IDO (-1 and/or−2) inhibition may be beneficial for SLE patients suffering from glomerulonephritis.

## Ethics Statement

All mice were maintained in the Biological Research Unit at the Lerner Research Institute, in accordance with Cleveland Clinic Foundation Animal Research Committee guidelines. Animal studies were approved by the Institutional Animal Care and Use Committee of the Lerner Research Institute of the Cleveland Clinic Foundation and conducted in compliance with guidelines issued by the National Institutes of Health.

## Author Contributions

LD performed all live experiments including genotyping, 1-d-MT treatments and immunization, all flow cytometry analyses, all ELISAs, and helped write the manuscript. JL performed IF staining of spleens and kidneys, and helped write the manuscript. LH performed the IDO enzymatic assay. TC performed IDO Western Blotting. AM collaborated and guided studies investigating IDO expression and enzymatic activity. TJ guided all animal experiments and wrote the manuscript.

### Conflict of Interest Statement

AM receives licensing and royalty income from NewLink Genetics Inc. The remaining authors declare that the research was conducted in the absence of any commercial or financial relationships that could be construed as a potential conflict of interest.
