# Supplementary material for: Limited Effect of Indolamine 2,3-Dioxygenase Expression and Enzymatic Activity on Lupus-Like Disease in B6.Nba2 Mice
Source: Front Immunol. 2019 Aug 27;10:2017. doi: 10.3389/fimmu.2019.02017 (PMC6727869; doi:10.3389/fimmu.2019.02017)
Supplement: Supplementary file 1 [file Table_1.pdf]

Supplemental Table 1: Splenic cellular composition in B6, B6.Nba2 and IDO-manipulated B6.Nba2 mice.

	B6[1]	B6.Nba2[2]	B6.Nba2.IDO1$^{-/-}$[3]	B6.Nba2 + 1-D-MT[4]	B6.Nba2.IDO1$^{-/-}$ + 1-D-MT[5]
**Total Splenocytes in millions**	75 ± 8.6[6]	130 ± 15.6*	113 ± 11.8*	100 ± 8.16(*)	117 ± 23.1
**Dendritic Cells**					
**cDCs**	0.6 ± 0.09[6]	1.7 ± 0.20**	1.2 ± 0.07**,(#)	1.1 ± 0.10*,#	1.1 ± 0.32
**pDCs (all)**	0.6 ± 0.1	1.8 ± 0.27**	1.6 ± 0.28(#)	1.2 ± 0.32	1.5 ±0.57
**$CD19^+$ pDCs**	0.3 ± 0.06	1.2 ± 0.28*	1.3 ± 0.25*	0.7 ± 0.28	1.2 ± 0.52
**$SiglecH^+$ pDCs**	0.2 ± 0.06	0.3 ± 0.05	0.2 ± 0.04	0.2 ± 0.02(#)	0.3 ± 0.08
**T cells**					
**$CD8^+$**	10.6 ± 1.41	15.1 ± 1.73(*)	11.8 ± 1.79	13.0 ± 1.11	12.5 ± 1.11
**$CD4^+$ (all)**	17.3 ± 3.65	29.0 ± 4.65(*)	22.9 ± 4.35	23.2 ± 3.21	20.9 ± 2.62
**$Foxp3^+CD4^+$ Treg**	0.89 ±0.27	2.0 ± 0.30*	1.5 ± 0..23	1.4 ± 0.26	1.5 ± 0.32
**Naïve $CD4^+$ $CD62L^{hi}CD44^{low}$**	12.3 ±2.52	13.9 ± 2.47	10.3 ± 2.87	13.0 ± 1.99	9.0 ± 0.99
**Eff-mem $CD4^+$ $CD62L^{low}CD44^{hi}$**	2.1 ±0.44	8.3 ± 1.68**	6.8 ± 0.86**	4.9 ± 0.83*,(#)	6.8 ± 1.51(*)
**$CD69^+CD4^+$**	1.3 ± 0.28	3.9 ±0.81*	3.3 ±0.61*	2.3 ±0.41(*)	3.1 ±0.68(*)
**Myeloid cells**					
**$Gr1^+CD11b^+$**	2.1 ± 0.29	3.3 ± 0.37*	5.6 ± 1.85	2.5 ± 0.22	3.5 ± 1.43
**$F4/80^+CD11b^+$**	0.9 ± 0.19	0.8 ± 0.15	0.7 ± 0.34	0.5 ± 0.06	0.6 ± 0.19
**$SignR1^+$ MΦ**	0.4 ± 0.06	0.3 ± 0.03	0.3 ± 0.02	0.3 ± 0.06	0.2 ± 0.05
**B cells**					
**All B cells**	36.5 ± 6.70	64.4 ± 8.02*	56.7 ± 7.75	49.8 ± 4.77	65.0 ± 16.6
**$CD69^+B220^+$**	0.1 ± 0.02	0.3 ± 0.06*	0.3 ± 0.02**	0.2 ± 0.03(*)	0.2 ± 0.05
**GC B cells**	0.40 ± 0.12	2.38 ± 0.38**	1.70 ± 0.14**	1.59 ± 0.41*	1.62 ± 0.32*
**$CD138^+B220^{low}$ Plasma cells**	0.06 ± 0.008	1.0 ± 0.28*	1.1 ± 0.15**	0.55 ± 0.15*	0.66 ± 0.29

[1] n = 3; [2] n = 8; [3] n = 4; [4] n = 7; [5] n = 4. [6] AVG ± SEM, numbers expressed in millions.
* $p < 0.05$; ** $p < 0.01$; *** $p < 0.001$; (*) p = 0.06-0.09: versus B6. # $p \leq 0.05$; (#) p = 0.06-0.09: versus B6.Nba2
