# Supplementary figures and images for: Limited Effect of Indolamine 2,3-Dioxygenase Expression and Enzymatic Activity on Lupus-Like Disease in B6.Nba2 Mice
Source: Front Immunol. 2019 Aug 27;10:2017. doi: 10.3389/fimmu.2019.02017 (PMC6727869; doi:10.3389/fimmu.2019.02017)

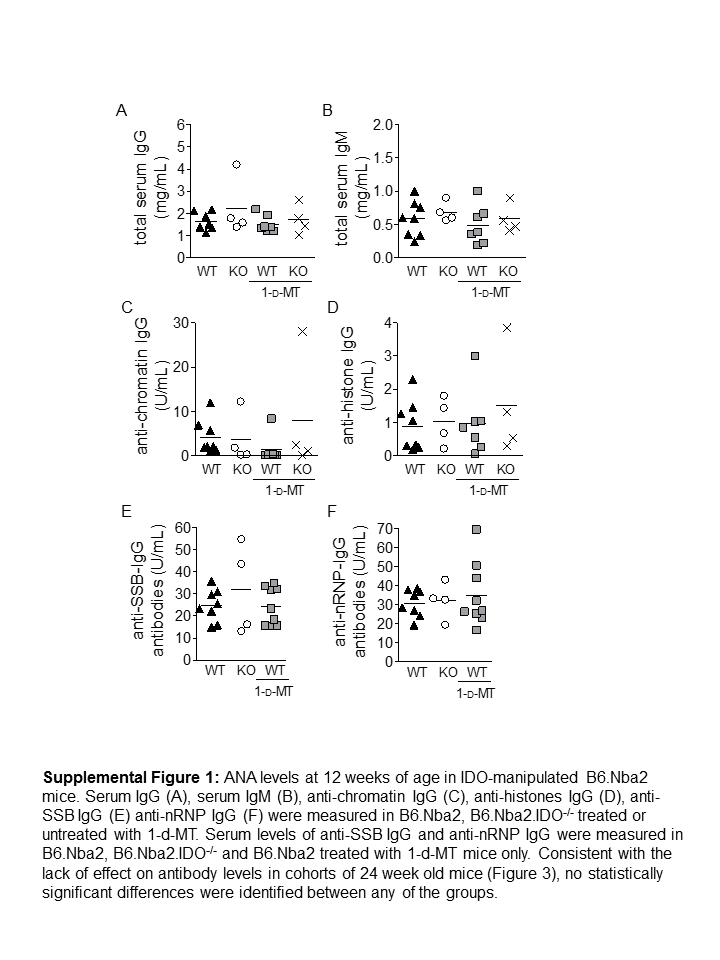

Supplement: Supplementary file 2 [file Image_1.TIF]
